# Antimicrobial, Immunomodulatory and Anti-Inflammatory Potential of Liposomal Thymoquinone: Implications in the Treatment of Bacterial Pneumonia in Immunocompromised Mice

**DOI:** 10.3390/biomedicines9111673

**Published:** 2021-11-12

**Authors:** Khaled S. Allemailem, Ahmad Almatroudi, Faris Alrumaihi, Aseel Aljaghwani, Abdullah M. Alnuqaydan, Habibullah Khalilullah, Hina Younus, Asmaa M. El-Kady, Fahad M. Aldakheel, Amjad Ali Khan, Arif Khan, Masood Alam Khan

**Affiliations:** 1 Department of Medical Laboratories, College of Applied Medical Sciences, Qassim University, Buraydah 51452, Saudi Arabia; K.allemailem@qu.edu.sa (K.S.A.); aamtrody@qu.edu.sa (A.A.); f_alrumaihi@qu.edu.sa (F.A.); aseel708@hotmail.com (A.A.); 2 Department of Medical Biotechnology, College of Applied Medical Sciences, Qassim University, Buraydah 51452, Saudi Arabia; ami.alnuqaydan@qu.edu.sa; 3 Department of Pharmaceutical Chemistry and Pharmacognosy, Unaizah College of Pharmacy, Qassim University, Unaizah 51911, Saudi Arabia; shabib79@gmail.com; 4 Interdisciplinary Biotechnology Unit, Aligarh Muslim University, Aligarh 202002, India; hinayounus@rediffmail.com; 5 Department of Medical Parasitology, Faculty of Medicine, South Valley University, Qena 83523, Egypt; asmaa.elkady@med.svu.edu.eg; 6 Department of Clinical Laboratory Sciences, College of Applied Medical Sciences, King Saud University, Riyadh 11564, Saudi Arabia; faldakheel@ksu.edu.sa; 7 Department of Basic Health Sciences, College of Applied Medical Sciences, Qassim University, Buraydah 51452, Saudi Arabia; akhan@qu.edu.sa (A.A.K.); 4140@qu.edu.sa (A.K.)

**Keywords:** *Acinetobacter baumannii*, liposomes, thymoquinone, pneumonia, immunocompromised mice

## Abstract

*Acinetobacter baumannii* has recently been increasing as an aggressive pathogen in immunocompromised persons. In the present study, we determined the in vitro antibacterial and anti-biofilm activity of thymoquinone (TQ) against *A. baumannii*. A liposomal formulation of TQ (Lip-TQ) was prepared and its therapeutic potential was investigated in the treatment of *A. baumannii* infection in immunocompromised mice. Leukopenia was induced in mice by injecting cyclophosphamide (CYP) at a dose of 200 mg/kg and the leukopenic mice were infected with 1 × 10^6^ CFUs of *A. baumannii*. The effectiveness of free TQ or Lip-TQ against *A. baumannii* infection was assessed by analyzing the survival rate and bacterial burden. Moreover, the efficacy of Lip-TQ was also studied by examining the systemic inflammatory markers and the histological changes in the lung tissues. The results showed that the mice in the group treated with Lip-TQ at a dose of 10 mg/kg exhibited a 60% survival rate on day 40 post-infection, whereas all the mice treated with free TQ at the same dose died within this duration. Likewise, the lowest bacterial burden was found in the lung tissue of mice treated with Lip-TQ (10 mg/kg). Besides, Lip-TQ treatment remarkably alleviated the infection-associated inflammation, oxidative stress, and histological changes in the lung tissues. Based on the findings of the present study, we recommend considering Lip-TQ as a valuable therapeutic formulation in the treatment of *A. baumannii*-associated pneumonia in immunocompromised subjects.

## 1. Introduction

In recent years, *Acinetobacter baumannii* infections have posed a massive challenge for clinicians due to its increasing prevalence in vulnerable persons [1,2]. The cells of the innate immune system, particularly neutrophils and macrophages, play a very important role in combating *A. baumannii* [3]. Immunocompromised mice demonstrated more severe pneumonia and an early impairment of the lung function [4]. In addition, neutropenia has been reported to increase the susceptibility of mice to *A. baumannii* [5]. Lately, *A. baumannii* has shown a remarkable degree of resistance to commonly used first-line antibiotics [6]. Currently, colistin and tigecycline are considered the most effective antibiotics in the treatment of the multidrug-resistant *A. baumannii*. However, some isolates of colistin-resistant *A. baumannii* have also emerged in different parts of the world [7]. *A. baumannii* adopts multiple mechanisms of drug resistance, including alteration of the target site, induction of drug-efflux pumps, and the formation of biofilms [8]. Biofilm formation is one of the strategies *A. baumannii* uses to render the drugs ineffective against the pathogen [9]. In addition, the biofilm is considered to play an important role in spreading *A. baumannii* infection through its formation on catheters, hospital surfaces, and other patient indwelling devices. 

In recent decades, the use of herbal medicines has been extensively investigated in the treatment of infectious diseases. Moreover, combinations of phytochemicals and antibiotics have been proved to be very effective against multidrug-resistant infections [10,11]. We earlier reviewed the properties of *Nigella sativa* and its main constituent thymoquinone (TQ) in the treatment of infectious diseases [12]. In addition to its antimicrobial activity, TQ exhibited anticancer, anti-inflammatory, antidiabetic, and antiasthmatic properties [13]. Despite having many beneficial properties, the poor solubility and bioavailability of TQ restricts its implications in the clinical setting. In order to increase its activity and bioavailability, nanoformulations of TQ have been prepared and investigated against many diseases [14]. In the present study, we prepared a formulation of liposomal TQ (Lip-TQ) and tested its activity in the treatment of *A. baumannii*-induced pneumonia in leukopenic mice. The results demonstrated that Lip-TQ was highly effective in the treatment of *A. baumannii*-induced pneumonia.

## 2. Materials and Methods

### 2.1. Materials

Phosphatidylcholine (PC), cholesterol (Chol), thymoquinone (TQ), and cyclophosphamide (CYP) were purchased from Sigma-Aldrich (St. Louis, MO, USA). Bacterial culture media, including Nutrient Agar, Nutrient broth, Tryptic Soya Agar and Tryptic Soya broth, were obtained from HiMedia Company (Mumbai, India). The kits for the analysis of cytokines, malondialdehyde (MDA), superoxide dismutase (SOD), and reduced glutathione (GSH) were purchased from Abcam (Cambridge, UK).

### 2.2. Acinetobacter baumannii

*A. baumannii* (ATCC 19606) was acquired from the American Type Culture Collection (ATCC, Manassas, VA, USA) and was maintained in Nutrient agar culture media plates.

### 2.3. Antimicrobial Susceptibility Testing

The minimum inhibitory concentration (MIC) of TQ was calculated by following the guidelines of the Clinical and Laboratory Standards Institute (CLSI) [15]. In order to determine MIC, TQ was taken in the concentration range from 0.125 to 128 µg/mL. The bacteria inoculum was cultured in nutrient broth, the cells were centrifuged, and the pellet was suspended in nutrient broth to 0.5 McFarland turbidity in the presence of the above-mentioned concentrations of TQ. The tubes containing the bacteria and TQ were incubated for 24 h. The MIC was considered the lowest concentration of TQ in the tube that did not show any turbidity of *A. baumannii* growth.

### 2.4. Bactericidal Activity of Free TQ or Lip-TQ on the Biofilm Formation and on the Preformed Biofilm by A. baumannii

The activity of TQ against the biofilm formation and preformed biofilm was analyzed as reported earlier [11]. *A. baumannii* was cultured in Tryptic Soya Broth (TSB). The bacterial cells at a density of 1 × 10^6^ CFUs/mL were placed in a 96-well plate and incubated for 24 h in the presence of free TQ or Lip-TQ at a concentration of 2 and 4 µg/mL. In order to assess the effect of the drug formulations against the preformed biofilm, the bacterial cells were placed in a 96-well plate and incubated for 24 h without any drug treatment. Keeping the preformed biofilm intact, fresh TSB containing free TQ or Lip-TQ (2 and 4 µg/mL) was added into the wells. The plate was incubated for 24 h at 37 °C, and washed with the sterile phosphate-buffered saline (PBS). The wells were dried, and 0.1% crystal violet solution was added to each well. The plate was washed, dried, and 100 µL of ethanol were added to each well to suspend the stain. Finally, the absorbance of the content was taken at 595 nm.

### 2.5. Formulation of TQ-Loaded Lipid Nanoparticles

The liposomal formulation of TQ was prepared and characterized as described earlier [16]. All the constituents, including PC, Chol, and TQ, were dissolved in a methanol and chloroform mixture (1:1 Vol/Vol) in a round-bottom flask. Methanol and chloroform were evaporated, and a thin film of the lipid and drug was formed that was hydrated to form a suspension. The suspension was sonicated and centrifuged to separate free TQ and Lip-TQ. The amount of TQ entrapped in liposomes was estimated by measuring the absorbance at 330 nm. The entrapment efficiency (EE) of TQ in liposomes was estimated by the following equation: % EE of TQ = (TQ entrapped in lipid nanoparticles/total TQ originally added) × 100. The percent EE of TQ in liposomes was calculated to be 90%.

### 2.6. Mice

Female Swiss mice (25–30 g) were taken from the animal house facility of the College of Pharmacy, King Saud University (KSU), Riyadh, Saudi Arabia. All experiments were performed according to the regulations of the animal ethics committee of the College of Applied Medical Sciences, Qassim University.

### 2.7. Induction of Leukopenia in Mice

Leukopenia was induced by injecting cyclophosphamide (CYP) at a dose 200 mg/kg through the intraperitoneal route [17]. After 4 days of CYP injection, the blood was collected from the mice to determine the leukopenia status.

### 2.8. Standardization of A. baumannii Infection in Leukopenic Mice

*A. baumannii* was cultured in TSB for 24 h at 37 °C. The bacterial cells were centrifuged, and the cell pellet was washed with PBS. In order to standardize the dose of in vivo infection, mice in various groups were infected with 5 × 10^5^, 1 × 10^6^, 5 × 10^6^, and 1 × 10^7^ CFUs of bacteria through the intravenous route. Each group contained 10 mice and the survival of the mice was observed for 10 days.

### 2.9. Infection of Leukopenic Mice with A. baumannii

After confirming the status of leukopenia and the standardization of the infection inoculum, each mouse was infected with 1 × 10^6^ CFUs of *A. baumannii* through the intravenous route.

### 2.10. Treatment of A. baumannii-Infected Mice with TQ Formulations

The efficacy of free TQ or Lip-TQ at doses of 5 and 10 mg/kg was tested in the treatment of *A. baumannii*-infected leukopenic mice. After 24 h, infected mice were treated with a daily dose of free TQ or Lip-TQ for 7 days. Mice were divided into six groups and each group contained 10 mice: (1) saline, (2) sham liposomes, (3) free TQ-5, (4) free TQ-10, (5) Lip-TQ-5, and (6) Lip-TQ-10. The mice were observed daily for a period of 40 days post infection.

### 2.11. Determination of Bacterial Load in the Lung Tissues

The efficacy of the therapy was evaluated by examining the load of *A. baumannii* in the lung tissues [16]. On day 7, three mice were randomly selected from each group and sacrificed to excise the lungs. Equally weighed parts of the lung tissues were homogenized in sterile PBS. After a suitable dilution, 100 μL of the homogenate were used for spreading on the nutrient agar plates. The culture plates were incubated for 24 h to detect the growth of bacteria. The numbers of bacterial CFUs were calculated by multiplying the dilution factor.

### 2.12. Determination of Inflammation Markers 

In order to evaluate the efficacy of TQ formulations in the treatment of *A. baumannii*-induced inflammation, the levels of C-reactive protein (CRP), IL-6, IL-1β, and TNF-α were measured in the blood of mice untreated or treated with TQ formulations [18].

### 2.13. Evaluation of the Status of Oxidative Stress in the Lungs

The status of oxidative stress was evaluated by analyzing the quantities of malondialdehyde (MDA), superoxide dismutase (SOD), and reduced glutathione (GSH) in lung tissue homogenate as described earlier [18]. In brief, a 100 mg piece of lung tissue was homogenized by using the lysis buffer. The tissue homogenate was centrifuged, and the supernatant was collected for the analysis of MDA, SOD, and GSH.

### 2.14. Histological Study of the Lung Tissue

The severity of infection-associated complications was assessed by histological study of the lung tissues of treated and untreated mice. The tissues were fixed with 10% neutral-buffered formalin solution. The paraffin-embedded blocks were prepared and sections of 5 μm thickness were sliced. The slides were stained with a Hematoxylin-Eosin (H&E) solution [18]. The tissues were studied under the light microscope to examine the pathological alterations in the tissues of the untreated or treated mice.

### 2.15. Statistical Analyses

Survival data are presented by a Kaplan–Meier curve and analyzed by the Log-rank Chi square test. The bacterial load, inflammation, and antioxidant parameters were analyzed by one-way ANOVA followed by the Turkey post-test using GraphPad Prism software, version 6.0 (La Jolla, CA, USA).

## 3. Results

### 3.1. TQ Shows Potent Activity against A. baumannii

The MIC of TQ was determined by monitoring the turbidity in the tubes containing *A. baumannii* and TQ. The MIC of TQ against *A. baumannii* was calculated to be 5 µg/mL. 

### 3.2. TQ Effectively Inhibited the Biofilm Formation and Eradicated the Preformed Biofilm 

**Figure 1:**
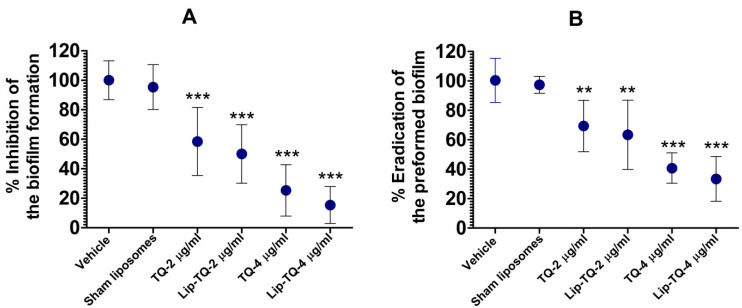
The effect of free TQ or Lip-TQ (2 and 4 µg/mL) against (**A**) biofilm formation and (**B**) preformed biofilm by *A. baumannii*. A *p* value <0.05 was considered to be significant. ** (*p* < 0.01), *** (*p* < 0.001) free TQ vs. vehicle treatment, Lip-TQ vs. sham liposomes treatment. The data are represented as the mean ± SD of three independent values. The data were analyzed by one-way ANOVA followed by the Turkey post-test.

Treatment with free TQ or Lip-TQ not only inhibited the formation of the biofilm, but also substantially eradicated the preformed biofilm (Figure 1A,B). Free TQ at a dose of 2 µg/mL caused 42% inhibition and at a dose of 4 µg/mL, it caused 75% inhibition in the biofilm formation as compared to the vehicle treatment (Figure 1A) (*p* < 0.001). Similarly, Lip-TQ at a dose of 2 µg/mL caused 50% inhibition, whereas at a dose of 4 µg/mL, it caused 85% inhibition (*p* < 0.001). However, no significant difference was observed between the treatments of free TQ and Lip-TQ at the comparable doses. The treatment with sham liposomes alone did not notably inhibit the biofilm formation as compared to the vehicle treatment. Interestingly, the treatment with free TQ or Lip-TQ was also effective against the preformed biofilm. Free TQ at a dose of 2 µg/mL eliminated 31% of the preformed biofilm, whereas at a dose 4 µg/mL, it eliminated 59% of the preformed biofilm as compared to the vehicle treatment (Figure 1B) (*p* < 0.01 and *p* < 0.001, respectively). Lip-TQ at the comparable doses eradicated 37% and 67% of the preformed biofilm (*p* < 0.01 and *p* < 0.001, respectively).

### 3.3. CYP Administration Induces Acute Leukopenia in Mice

**Figure 2:**
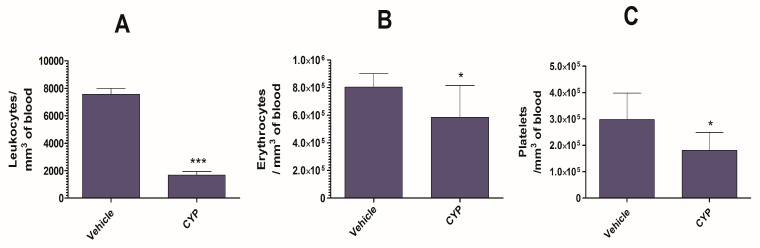
The administration of CYP causes a depletion of (**A**) leukocytes, (**B**) erythrocytes, and (**C**) blood platelets in mice. A *p* value <0.05 was considered to be significant. * (*p* < 0.05), *** (*p* < 0.001) Each bar represents the mean ± SD of three different values. The data were analyzed by one-way ANOVA followed by the Turkey post-test.

The administration of CYP resulted in a remarkable reduction in the numbers of leukocytes, erythrocytes, and blood platelets (Figure 2A–C). CYP-injected mice displayed leukocyte numbers of 1682 ± 247 per mm^3^ of blood, whereas vehicle-injected mice displayed leukocyte numbers of 7566 ± 419 (*p* < 0.001) (Figure 2A). In addition, CYP injection lowered the erythrocyte count from 804,900 ± 22,750 to 58,5700 ± 53,640 per mm^3^ of blood (Figure 2B) (*p* < 0.05). The administration of CYP also depleted the numbers of blood platelets in the mice (Figure 2C). Mice injected with CYP had a platelet count of 181,600 ± 15,700 per mm^3^, whereas vehicle-treated mice had a platelet count of 298,200 ± 23,110 per mm^3^ of blood (Figure 2C) (*p* < 0.05). 

### 3.4. Standardization of the Dose of A. baumannii in Immunocompetent and Immunocompromised Mice

**Figure 3:**
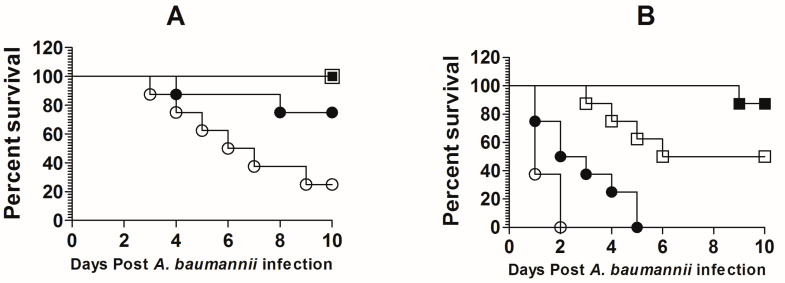
Standardization of the infectious dose of *A. baumannii* in mice. (**A**) Immunocompetent and (**B**) leukopenic mice were infected with various CFUs of *A. baumannii* and their survival was monitored for 10 days. (■) 5 × 10^5^, (☐) 1 × 10^6^, (⬤) 5 × 10^6^, (◯) 1 × 10^7^.

The dose of *A. baumannii* infection was standardized in the immunocompetent and immunocompromised mice in order to select the most appropriate infection inoculum. Immunocompetent mice infected with 1 × 10^7^ CFUs of *A. baumannii* showed a 20% survival rate, whereas the mice infected with 5 × 10^6^ CFUs showed 80% survival on day 10 post-infection (Figure 3A). Mice infected with 1 × 10^6^ and 5 × 10^5^ CFUs showed a 100% survival rate on day 10 post-infection. On the contrary, leukopenic mice demonstrated greater susceptibility to *A. baumannii* as the mice infected with 1 × 10^7^ CFUs of *A. baumannii* died within 2 days of infection. The leukopenic mice infected with 5 × 10^6^ CFUs of *A. baumannii* died by day 5 post-infection, whereas those infected with 1 × 10^6^ exhibited a 50% survival rate on day 10 post-infection (Figure 3B). Based on the findings of the survival data, a dose of 1 × 10^6^ cells of *A. baumannii* was chosen to infect the leukopenic mice in the future experiments.

### 3.5. Treatment with Lip-TQ, Not with Free TQ, Was Effective against A. baumannii in Leukopenic Mice

**Figure 4:**
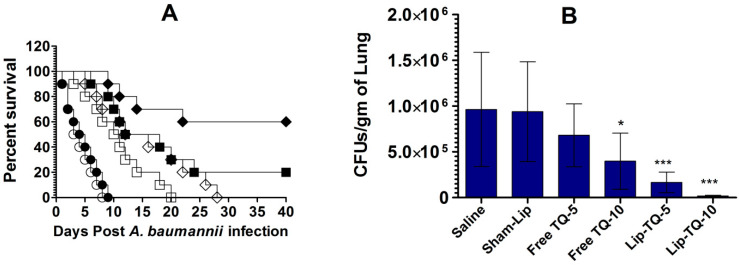
Treatment with Lip-TQ was highly effective against *A. baumannii* in the leukopenic mice. (**A**) Each mouse was intravenously infected with 1 × 10^6^ CFUs of *A. baumannii*. After 24 h, the infected mice were treated with a single daily dose of free TQ or Lip-TQ (5 and 10 mg/kg) for 7 days. Saline (◯), Sham-Lip (⬤), Free TQ-5 mg/kg (☐), Free TQ-10 mg/kg (◇), Lip-TQ-5 mg/kg (■), Lip-TQ-10 mg/kg (♦). Saline vs. Free TQ-5 mg/kg (*p* = 0.009), Sham-Lip vs. Lip-TQ-5 mg/kg (*p* < 0.0001), Saline vs. Free TQ-10 mg/kg (*p* = 0.0001), Sham-Lip vs. Lip-TQ-10 mg/kg (*p* < 0.0001), Free TQ-10 mg/kg vs. Lip-TQ-10 mg/kg (*p* = 0.0074). The survival data are presented by a Kaplan–Meier curve and analyzed by the Log-rank Chi square test. (**B**) On day 7 post-infection, 3 mice from each group were sacrificed and equally weighed pieces of the lung tissue were homogenized in sterile normal saline. After a proper dilution, the tissue homogenate was spread on the nutrient agar plates. The plates were incubated at 37 °C for 24 h. The CFUs were counted, and the total numbers were calculated by multiplying the dilution factor. The data are represented as the mean ± SD of three independent values. * (*p* < 0.05), *** (*p* < 0.001). The data were analyzed by one-way ANOVA followed by the Turkey post-test.

The therapeutic efficacy of TQ formulations was assessed against the systemic *A. baumannii* infection in the leukopenic mice. The outcomes demonstrated that the mice treated with free TQ at the doses of 5 and 10 mg/kg died before day 40 post-*A. baumannii* infection (Figure 4A). The median survival time (MST) of the mice treated with saline was 3.5 days, whereas the MST of mice treated with free TQ, at doses of 5 and 10 mg/kg, was found to be 10.5 and 14 days, respectively (Figure 4A). The mice in the group treated with Lip-TQ at a dose of 5 mg/kg showed a 20% survival rate, whereas those treated with 10 mg/kg had a 60% survival rate on day 40 post-infection (Figure 4A). Importantly, the treatment with Lip-TQ at a dose of 10 mg/kg exhibited superior efficacy as compared to the treatment with free TQ at the same dose (*p* = 0.0074).

The extent of *A. baumannii* infection was assessed by analyzing the bacterial load in the lung tissue of mice untreated or treated with TQ formulations (Figure 4B). The infected mice treated with saline had a bacterial load of 963,047 ± 251,379 CFUs/gm in the lung tissue. The treatment with free TQ at a dose of 5 mg/kg reduced the bacterial load to 681,437 ± 138,284 and the treatment at a dose of 10 mg/kg decreased the bacterial load to 396,945 ± 123,826 CFUs/gm. On the other hand, the mice treated with Lip-TQ at a dose of 5 mg/kg had a bacterial burden of 166,415 ± 45,598 CFUs/gm. Whereas, the mice treated with Lip-TQ at a dose of 10 mg/kg had a bacterial load of 17,703 ± 3423 CFUs/gm, which was significantly lower compared to the bacterial load of 938,660 ± 219,801 CFUs/gm in the lung tissue of the sham-lip-treated mice (*p* < 0.001). In order to confirm that the survived mice were free of infection, the bacterial load was assessed in the lung of the surviving mice on day 40 post-infection. The results demonstrated that the surviving mice did not have any bacterial load in their lung tissues. 

### 3.6. Lip-TQ Treatment Alleviated the Levels of CRP, IL-6, IL-1β, and TNF-α in the Systemic Circulation of A. baumannii-Infected Mice

**Figure 5:**
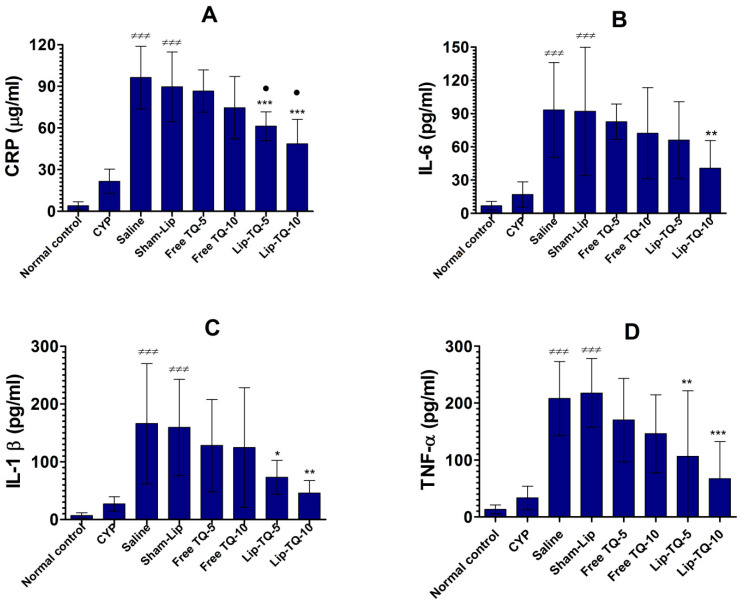
Treatment with Lip-TQ alleviated the systemic inflammation in *A. baumannii*-infected mice. (**A**) CRP. ^≠≠≠^ (*p* < 0.001) CYP vs. Saline or Sham-Lip, *** (*p* < 0.001) Sham-Lip vs. Lip-TQ-5 or Lip-TQ-10, ^⬤^ (*p* < 0.05) Free TQ-5 vs. Lip-TQ-5 or Free TQ-10 vs. Lip-TQ-10. (**B**) IL-6. ^≠≠≠^ (*p* < 0.001) CYP vs. Saline or Sham-Lip, ** (*p* < 0.01) Sham-Lip vs. Lip-TQ-10. (**C**) IL-1β. (*p* < 0.001) CYP vs. Saline or Sham-Lip, * (*p* < 0.05) Sham-Lip vs. Lip-TQ-5, ^≠≠≠^ (*p* < 0.001) CYP vs. Saline or Sham-Lip, ** (*p* < 0.01) Sham-Lip vs. Lip-TQ-10. (**D**) TNF-α. ^≠≠≠^ (*p* < 0.001) CYP vs. Saline or Sham-Lip, Sham-Lip vs. Lip-TQ-5, ** (*p* < 0.01), *** (*p* < 0.001) Sham-Lip vs. Lip-TQ-10. The data are represented as the mean ± SD of three independent values. The data were analyzed by one-way ANOVA followed by the Turkey post-test.

CRP is one of the commonest biomarkers that should be analyzed to diagnose the extent of infection-associated inflammation. The CRP level increased to 96.33 ± 9.1 µg/mL in the mice of the saline-treated group in comparison to the CRP level of 4.1 ± 1.81 µg/mL in normal mice (Figure 5A) (*p* < 0.001). Free TQ at doses of 5 and 10 mg/kg did not significantly reduce the CRP level in the treated mice (*p* > 0.05) whereas the treatment with the same doses of Lip-TQ decreased the CRP level to 61.33 ± 4.2 and 48.67 ± 7.1 µg/mL, which were significantly lower compared to the CRP level of 89.67 ± 10 µg/mL in the sham-lip-treated mice (*p* < 0.001). Interestingly, the treatment with Lip-TQ significantly reduced the CRP level compared to the treatment with free TQ at the respective doses (*p* < 0.05).

Proinflammatory cytokines, such as IL-6, IL-1β, and TNF-α, play an essential role in infection-induced inflammatory responses. The amount of IL-6 was increased to 93.33 ± 17.24 pg/mL in infected mice treated with saline as compared to its level of 6.8 ± 1.58 pg/mL in normal mice (*p* < 0.001) (Figure 5B). However, free TQ at the doses of 5 and 10 mg/kg did not substantially alleviate the IL-6 level in the treated mice (Figure 5B). Instead, Lip-TQ at a dose of 10 mg/kg significantly reduced the level of IL-6 in the treated mice (*p* < 0.05). Similarly, IL-1β production was also highly elevated in the systemic circulation of *A. baumanni*-infected mice (Figure 5C). The IL-1β level was increased to 166 ± 41.9 pg/mL in the blood of saline-treated mice as compared to an IL-1β level of 7 ± 2 pg/mL in normal mice (*p* < 0.001). The mice treated with Lip-TQ, at doses of 5 and 10 mg/kg, decreased the IL-1β level to 73.33 ± 11.72 and 46 ± 8.7 pg/mL, respectively (*p* < 0.05 and *p* < 0.01, respectively). Similar to the IL-6 and IL-1β levels, TNF-α was also raised in the blood of *A. baumanni*-infected mice. Infected mice treated with saline had a TNF-α level of 208 ± 26.23 pg/mL compared to its level of 33 ± 8.3 pg/mL in CYP-injected mice not infected with *A. baumannii*, respectively (Figure 5D) (*p* < 0.001). The administration of Lip-TQ (5 and 10 mg/kg) decreased the TNF-α level to 106.7 ± 46 and 67.33 ± 26 pg/mL, respectively (*p* < 0.01 and *p* < 0.001, respectively).

### 3.7. Lip-TQ Treatment Improved the Status of Oxidative Stress in A. baumannii-Infected Mice

**Figure 6:**
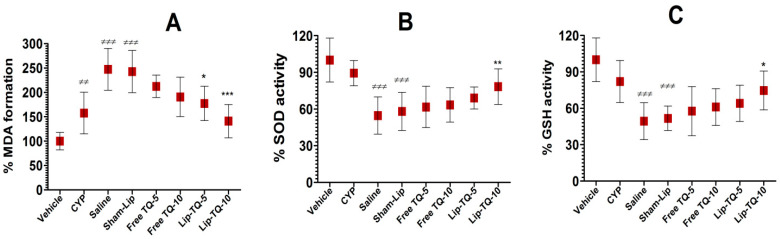
Lip-TQ therapy decreased the status of oxidative stress in the lung tissues. (**A**) MDA. ^≠≠^ (*p* < 0.01) Saline vs. CYP, ^≠≠≠^ (*p* < 0.001) CYP vs. Saline or Sham-Lip, * (*p* < 0.05) Sham-Lip vs. Lip-TQ-5, *** (*p* < 0.001) Sham-Lip vs. Lip-TQ-10. (**B**) SOD. ^≠≠≠^ (*p* < 0.001) CYP vs. Saline or Sham-Lip, ** (*p* < 0.01) Sham-Lip vs. Lip-TQ-10. (**C**) GSH. ^≠≠≠^ (*p* < 0.001) CYP vs. Saline or Sham-Lip, * (*p* < 0.05) Sham-Lip vs. Lip-TQ-10. The data are represented as the mean ± SD of three independent values. The data were analyzed by one-way ANOVA followed by the Turkey post-test.

MDA is a major product of lipid peroxidation that is elevated in the condition of oxidative stress. The formation of MDA in the lung tissues of CYP-injected mice was increased to 158% compared to the MDA level in normal mice (Figure 6A) (*p*< 0.01). It was further increased to 247% in *A. baumannii*-infected mice that were not treated with any drug formulation (*p* < 0.001). However, the treatment with Lip-TQ at a dose of 5 mg/kg decreased the formation of MDA to 177%, whereas the same formulation at a dose of 10 mg/kg reduced MDA formation to 141% compared to its level in normal mice (*p* < 0.05 and *p* < 0.001, respectively).

The results of the present study demonstrated that CYP-injected mice had reduced levels of SOD and GSH, which were further depleted in *A. baumannii*-infected mice (Figure 6B,C). The SOD activity was found to be 89.3% in the blood of CYP-injected mice, which was further reduced to 54.2% after infection with *A. baumannii* (Figure 6B). However, the treatments with free TQ at doses of 5 and 10 mg/kg did not induce any significant recovery of the SOD activity (Figure 6B). On the other hand, treatment with Lip-TQ at a dose of 10 mg/kg, not at 5 mg/kg, recovered the SOD activity to 78% (*p* < 0.01). Like the SOD activity, the activity of GSH in the blood of CYP-injected mice was reduced to 82% as compared to the GSH level in normal control mice (Figure 6C). *A. baumannii* infection induced a further drop in the GSH activity to 49.3% in CYP-injected mice (*p* < 0.001). Notably, the treatment with Lip-TQ at a dose of 10 mg/kg induced the recovery of the GSH activity to 74.6%, which was significantly higher than the GSH activity in the sham-lip-treated mice (*p* < 0.05). 

### 3.8. Lip-TQ Reversed Infection-Induced Pathological Changes in the Lung Tissues 

**Figure 7:**
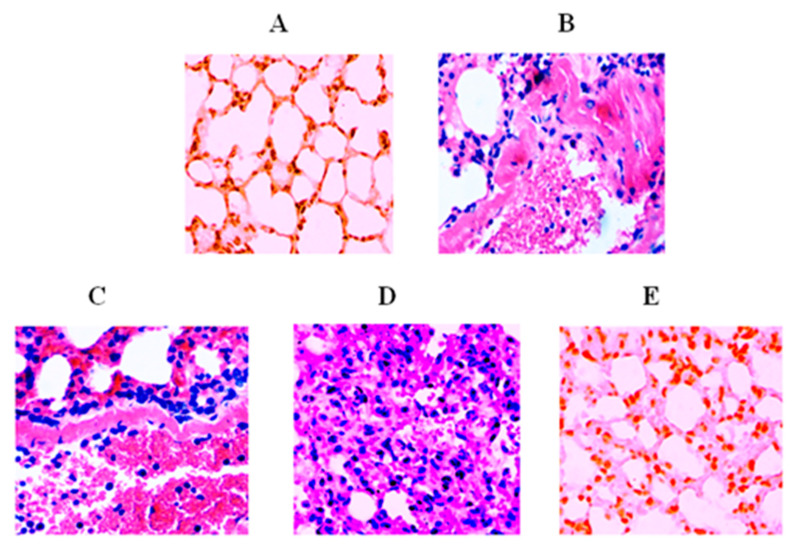
Treatment with Lip-TQ alleviated *A. baumannii*-associated pathological alterations in the lung tissues of infected mice. Histological study of the lung tissues of mice in the following groups: (**A**) uninfected normal control, (**B**) infected saline treatment, (**C**) infected sham liposome treatment, (**D**) infected free TQ-10 mg/kg treatment, and (**E**) infected Lip-TQ-10 mg/kg treatment. The amplification factor was 200×.

The histological examination of the lung tissues of *A. baumannii*-infected mice treated with saline or sham-lip revealed greater infiltration of inflammatory cells compared to the lung tissues from normal mice (Figure 7A–C). Moreover, extensive amounts of hemorrhage, fibrosis, congestion, and airway wall thickening were also observed in the lung tissues of *A. baumannii*-infected mice treated with saline or sham-lip (Figure 7B,C). The treatment with free TQ at a dose of 10 mg/kg moderately improved the status of inflammation and thickening of the airway in *A. baumannii*-infected mice (Figure 7D). Contrarily, Lip-TQ administration at a dose of 10 mg/kg effectively reversed the pathological changes in the lung tissues of *A. baumannii*-infected mice (Figure 7E). 

## 4. Discussion

*A. baumannii* has recently emerged as a pathogen of big concern, particularly in immunocompromised persons because an active immune system mediates a protective immune response against *A. baumannii* [3,19]. *A. baumannii* infection results in the recruitment of neutrophils to the infection site in order to combat the bacteria by releasing multiple molecules, including myeloperoxidase, reactive oxygen species (ROS), β-defensins, cytokines, and chemokines [3]. Garcia-Patino et. al. extensively reviewed the important role of neutrophils and other immune cells against *A. baumannii* infection [20]. In conformity, the results of the present study showed that neutrophils have a protective role against *A. baumannii* because leukopenic mice showed greater susceptibility to infection as compared to immunocompetent mice. The current strain of *A. baumannii* (ATCC 19606) has been reported to be resistant to a broad spectrum of antibiotics, including ampicillin, chloramphenicol, ceftriaxone, cexotaxime, penicillin, cefoxitin, and cephalothin [6]. In this context, the results of the current study are very encouraging because TQ demonstrated a very potent antimicrobial activity against the current multi-drug-resistant *A. baumannii*. Biofilm formation contributes to the virulence and the phenomenon of drug resistance in pathogens. The ability of biofilm formation of *A. baumannii* depends on its outer membrane protein A (*omp*A) and pili, which mediate the attachment of the biofilm to the surface [9]. TQ has earlier been shown to inhibit the formation of the biofilm by *Listeria monocytogenes*, *Staphylococcus aureus*, *Staphylococcus epidermidis*, *Klebsiella pneumoniae*, *Shigella flexneri*, *Candida albicans*, and *Candida glabrata* [21,22,23,24,25]. In agreement with the earlier reports, the results of the present study demonstrated that TQ amazingly inhibited biofilm formation and moreover, it also substantially eradicated the preformed biofilm by *A. baumannii*. 

Despite possessing a wide range of therapeutic properties, the clinical use of TQ has been restricted owing to its meager solubility and bioavailability. The incorporation of TQ in various drug delivery systems has augmented the therapeutic efficacy of the drug [14,16]. The results of the present study demonstrated greater activity of TQ in lipid nanoparticle formulation because *A. baumannii*-infected leukopenic mice treated with Lip-TQ (10 mg/kg) had a 60% survival rate on day 40 post-infection, whereas the mice in the group treated with free TQ at the same dose died before day 40 post-infection. The outcome of the survival data was also supported by the bacterial load data, which is an indicator of the severity of the infection. Like the survival data, the bacterial load was found to be the lowest in the lung tissues of mice treated with Lip-TQ at a dose of 10 mg/kg. The greater activity of Lip-TQ against *A. baumannnii* may be attributed to the higher bioavailability of the drug when administered in the liposomal form.

Greater secretion of proinflammatory cytokines, including IL-1β, IL-6, and TNF-α, has been reported in response to *A. baumannii* infection [26]. *A. baumannii* infection has induced the activation of NLRP3 inflammasome, which contributed to the production of IL-1β and the pathogenesis of the lungs [27]. The pathological role of IL-1β is supported by the finding that IL-1R-deficient mice infected with *A. baumannii* had reduced complications in their lung tissues [27]. In an earlier report, TQ was shown to inhibit the lipopolysaccharide (LPS)-induced secretion of IL-1β and restored pulmonary vascular functioning in an animal model [28]. The results of the present study demonstrated that the treatment of *A. baumannii*-infected mice with Lip-TQ resulted in a remarkable reduction in the level of IL-1β in the systemic circulation. Another cytokine IL-6 has also been found to be elevated in the conditions of inflammatory diseases [29]. An increased level of IL-6 was reported in chronic obstructive pulmonary disease (COPD) and is thought to be an indicator of poor clinical outcome in COPD patients [30]. A higher level of IL-6 has also been shown to play an important role in infection-associated inflammation [31]. Shaterzadeh-Yazdi et al. demonstrated that LPS induced the secretion of IL-6, whereas TQ inhibited the LPS-induced production of IL-6 by macrophages [31]. The results of the current study demonstrated that treatment with Lip-TQ significantly lowered the secretion of IL-6 in *A. baumannii*-infected mice. Similar to IL-1β and IL-6, TNF-α plays a critical role in *A. baumannii*-induced inflammation in infected mice [32]. The TNF-α level was remarkably elevated in the systemic circulation of *A. baumannii*-infected mice. Interestingly, treatment with Lip-TQ significantly decreased the level of TNF-α in *A. baumannii*-infected mice. 

CYP administration has been shown to induce a state of oxidative stress through the depletion of antioxidant enzymes, including SOD, GSH, and catalase [33]. Moreover, *A. baumannii* infection elevates the status of oxidative stress and proinflammatory cytokines, which induce cell death in the lung epithelial tissue [34]. Interestingly, the inhibition of TNF-α and interleukin-6 prevented infection-induced cell death. Furthermore, *A. baumannii* infection resulted in elevated MDA formation and decreased the amounts of SOD and GSH. Since TQ possesses antioxidant activity, treatment with Lip-TQ reversed the elevated parameters of oxidative stress in *A. baumannii*-infected mice. We earlier showed that the influx of inflammatory cells contributes to congestion and inflammation in the lung tissues of OVA-induced allergic asthmatic mice [18]. *A. baumannii* infection induces an influx of neutrophils and macrophages, which hugely contributes to inflammation in the lung tissues. The data in the present study suggested that Lip-TQ effectively alleviated the severity of inflammation and congestion in the lung tissues. It suggests that Lip-TQ may be explored as a promising therapeutic agent to cure *A. baumannii* infection and associated inflammatory complications. 

Concisely, Lip-TQ showed potent antibacterial activity against *A. baumannii* in a murine model. The superior activity of Lip-TQ was supported by the data of the survival rate and bacterial load in the treated mice. Furthermore, treatment with Lip-TQ not only reduced the levels of the systemic inflammatory markers, but also restored the status of MDA, SOD, and GSH close to the normal level. Interestingly, Lip-TQ alleviated the influx of inflammatory cells, congestion, and fibrosis in the lung tissues of treated mice. Together, these findings indicate that Lip-TQ may prove to be a very promising formulation in the treatment of *A. baumannii* infection and associated complications in immunocompromised subjects. 

## Data Availability

All relevant data have been provided within the manuscript. There are no supporting files and no data was held.
